# Supplementary material for: Warming Scenarios and Phytophthora cinnamomi Infection in Chestnut (Castanea sativa Mill.)
Source: Plants (Basel). 2023 Jan 26;12(3):556. doi: 10.3390/plants12030556 (PMC9921032; doi:10.3390/plants12030556)
Supplement: Supplementary file 1 [file plants-12-00556-s001.zip › plants-2161745-supplementary.pdf]

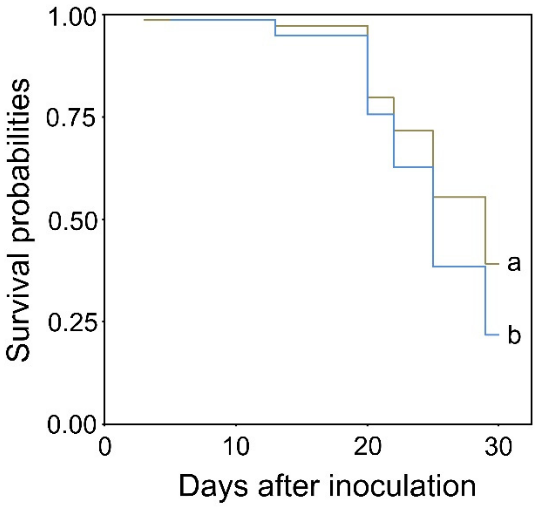

Figure S1: Survival probabilities of *Castanea sativa* seedlings from two mother trees (family 1 in blue and family 4 in brown) inoculated at day 0 with *Phytophthora cinnamomi*. Global log-rank test was significant at $p = 0.041$. Different letters indicate significant differences between survival curves ($p < 0.05$).

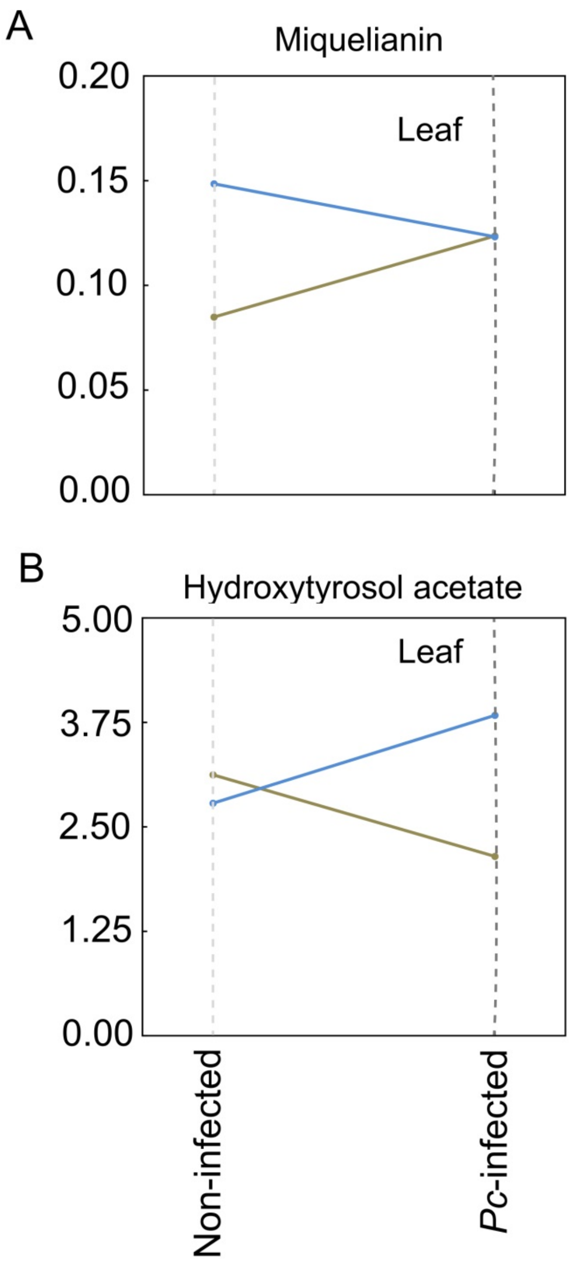

Figure S2: Phenolic compounds of *Castanea sativa* seedlings from two mother trees (family 1 in blue and family 4 in brown) that differently changed their contents in response to *Phytophthora cinnamomi* (significant *Pc* × mother tree interactions in Table 4; $p < 0.05$).

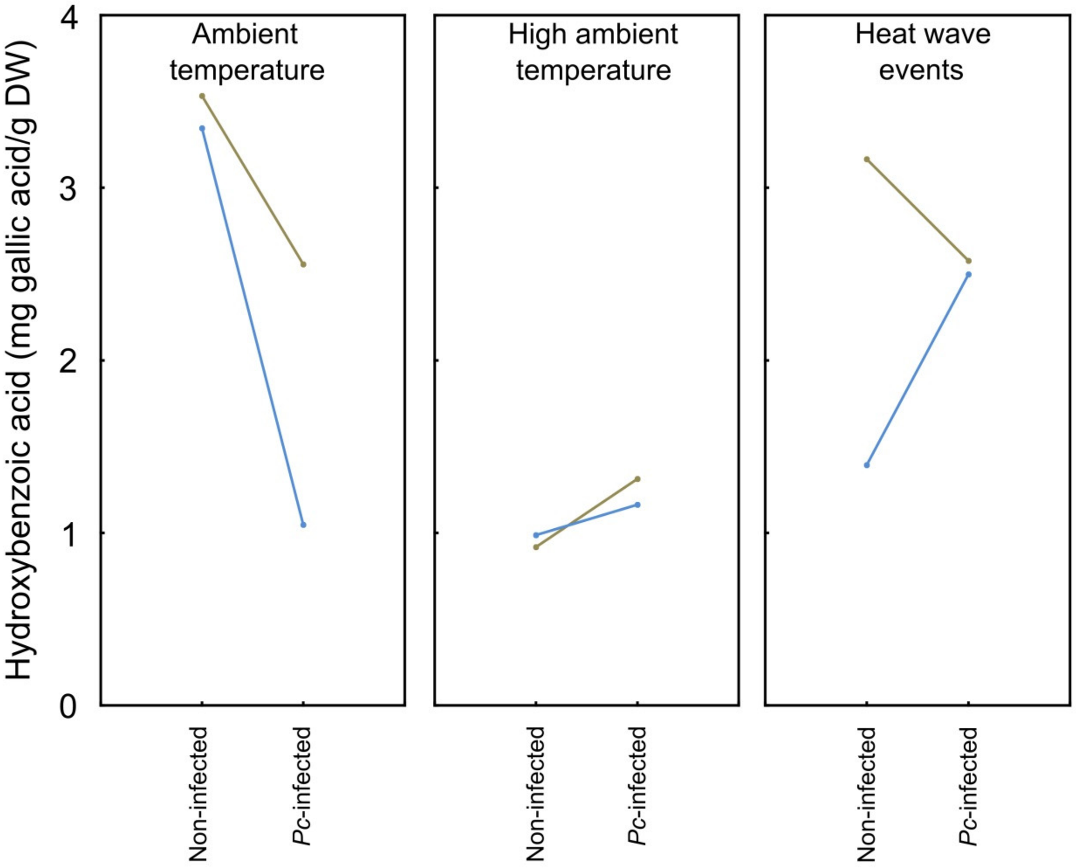

Figure S3: Phenolic compound of *Castanea sativa* seedlings from two mother trees (family 1 in blue and family 4 in brown) that differently changed it content in response to *Phytophthora cinnamomi* and the scenario experienced by plants before inoculation (significant *Pc* × S × mother tree interaction in Table 4; $p < 0.05$).
